# Unveiling the Threat to Vulture Diversity: A Comprehensive Ethno‐Ornithological Study Uncovers Regional Trade Effects in Côte d'Ivoire

**DOI:** 10.1002/ece3.70740

**Published:** 2024-12-23

**Authors:** Asso Armel Asso, Volker Salewski, N'golo Abdoulaye Koné

**Affiliations:** ^1^ Université Nangui ABROGOUA, UFR Sciences de la Nature (UFR SN), Laboratoire d'Écologie et de Développement Durable (LEDD) Abidjan Côte d'Ivoire; ^2^ Station de Recherche en Écologie du Parc National de la Comoé Abidjan Côte d'Ivoire; ^3^ Michael‐Otto‐Institut im NABU Bergenhusen Germany

**Keywords:** conservation, International Trade, magico‐traditional medicine, scavengers, stalls, West Africa

## Abstract

In recent decades, African vulture populations have experienced a distressing decline, with certain species plummeting by over 90%. This decline can largely be attributed to various human‐induced pressures. In West Africa, the trade of vultures for magico‐traditional medicine stands out as a significant threat. However, there remains a dearth of knowledge regarding the extent and economic ramifications of poaching and its associated trade on the biodiversity of these raptors in Côte d'Ivoire. This study sets out to gauge the scope of vulture trade and trafficking in Côte d'Ivoire, as well as its national and subregional repercussions, while also scrutinizing the potential constraints such activity imposes on the biodiversity of these raptors. Ethno‐ornithological investigations were conducted across 24 cities in Côte d'Ivoire, delving into the markets where various animals are showcased. The aim was to analyze the challenges stemming from the demand for vultures for magico‐traditional medicinal purposes and to examine the economic values entwined with poaching and trading these vultures. The findings illuminate the existence of a network dedicated to poaching and trading vultures in Côte d'Ivoire, driven by robust demand and regularly supplied by local and regional sources from neighboring countries. Predominantly, the Hooded Vulture ( *Necrosyrtes monachus* ) emerges as the most frequently encountered species on the market. In response to this imminent threat, it is advocated that national and regional awareness campaigns be undertaken to bolster enforcement of pertinent wildlife protection laws, particularly concerning vultures. Furthermore, fostering enhanced cooperation among West African governments is advised to avert the extinction of these species.

## Graphical abstract

In recent decades, African vulture populations have drastically declined, with some species decreasing by over 90%, largely due to human activities. In Côte d'Ivoire, the trade of vultures for magico‐traditional medicine poses a significant threat, with the Hooded Vulture ( *Necrosyrtes monachus* ) being the most commonly traded species. This study highlights the need for national and regional awareness campaigns and stronger cooperation among West African governments to protect vultures from extinction.

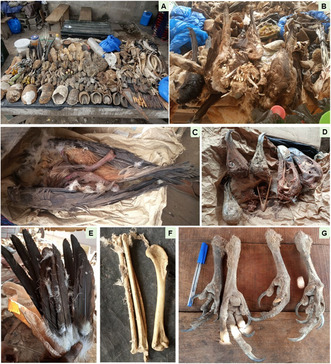

## 1 Introduction

Vultures stand as emblematic figures within the protected expanses of savannah ecosystems. Their morphologies, finely honed to suit their dietary habits, as extensively documented (Rich 1983; Sibley and Halquist 1990; Mundy et al. 1992; Donázar 1993; Hertel 1994; Hockey, Dean, and Ryan, 2005; Dupont 2011), bestow upon them a pivotal role in provisioning vital ecosystem services. These services include organic matter recycling, and the containment of potentially perilous diseases among other wildlife, humans and livestock alike. All these services contribute to the natural regulation function (Mundy et al. 1992; Morin and Findlay, 2001; Şekercioğlu et al. 2004; Craig, Thomson & santangeli, 2018; Frank and Sudarshan 2024). The significance of these services is notably contingent upon cultural and societal contexts (Dupont 2011), as exemplified by Markandya et al. (2008) delineation of the manifold benefits vultures afford in India, in particular through the reduction in the spread of rabies and the reduction in the mortality rate of organisms (Markandya et al. 2008; Ogada et al. 2012; Frank and Sudarshan 2024).

Yet, the past few decades have borne witness to precipitous declines in vulture populations across Africa, with West Africa emerging as a focal point of concern (Rondeau and Thiollay, 2004; Thiollay 2001, 2006; Ogada and Buij 2011; Ogada et al. 2016a; Jallow et al. 2022). Thiollay (1985) was among the earliest to signal this alarming trend, citing notable declines in Hooded Vultures ( *Necrosyrtes monachus* Temminck, 1823) within Côte d'Ivoire. Subsequent investigations substantiated these stark reductions across various West African countries (Thiollay 2006). Subsequent studies (Ogada and Buij 2011; Ogada et al. 2016a; Murn et al. 2016) have highlighted a general decline in the diversity and abundance of vultures, with a particularly alarming impact on the White‐headed Vulture species ( *Trigonoceps occipitalis* Burchell, 1824).

In West Africa, vultures have all but vanished from urban landscapes, while their survival within savannah habitats hangs precariously in the balance (Mullié et al. 2017; Safford et al. 2019). The underlying causes of this widespread decline are manifold, spanning deliberate and inadvertent poisoning, illegal hunting, habitat degradation, dwindling food sources and nesting grounds, as well as the perils of electrocution and collisions with power lines. Chief among these threats in the region is the exploitation of vulture parts for magico‐traditional medicinal practices (Cocker 2000; Nikolaus 2011; Saidu and Buij 2013; Gbogbo, Roberts, and Awotwe‐Pratt 2016; Williams et al. 2021), a phenomenon observed across several countries including Benin (Adjakpa, Tchabi, and Ogouvide 2002), Burkina Faso (Daboné et al. 2022), Ghana (Gbogbo, Roberts, and Awotwe‐Pratt 2016; Deikumah 2020) and Nigeria (Awoyemi 2014; Williams et al. 2021). Indeed, vultures or their derivatives are evidently highly prized, with indications of international trade permeating West Africa (Ogada and Buij 2011; Buij et al. 2016; Daboné et al. 2022).

Nevertheless, comprehensive understanding regarding the scale and ecological ramifications of vulture poaching and trade within the biodiversity tapestry of Côte d'Ivoire remains elusive. Furthermore, a dearth of scientific data impedes effective mitigation efforts against these looming threats. Notably, there exists a paucity of systematic inquiries into the markets of Côte d'Ivoire, where the Comoé National Park (CNP), situated in the country's northeast, stands as one of the last bastions for vultures in West Africa (Salewski 2017). Thus, the imperative to identify illicit poaching pressures fueled by the demand for vulture parts in magico‐traditional medicine, alongside quantifying the monetary worth of poaching and trade, is paramount, underscoring potential reverberations on the raptors' biodiversity of Côte d'Ivoire. Here, we present the results of a study that aimed to close this knowledge gap by investigating trade circuit with respect to vultures with a country‐wide market analysis.

### 1.1 Study Area

This investigation was conducted across the expanse of Côte d'Ivoire, a West African country situated between the longitudes 2°30′ and 8°30′ West and the latitudes 4°30′ and 10°30′ North.

The population of Côte d'Ivoire is estimated to be 29,389,350 individuals according to the latest general population and housing census, boasting a linguistic composition comprising 63 distinct ethnic groups (RGPH 2021). This demographic mosaic includes adherents of Islam (42.5%), Christianity (39.8%), animism (2.2%) and other faiths (0.7%), each contributing to the rich cultural variation spanning from the northern reaches to the southern expanses of the country (RGPH 2021). Notably, the population encompasses several prominent ethnic enclaves, among which are the Akans, the Gours, the northern Mandés, the Krous and the southern Mandés.

Côte d'Ivoire experiences a predominantly tropical climate, occupying the liminal space between the moist equatorial climate and the arid tropical climate (Goula et al. 2007; Jourda et al. 2015). The nation manifests four distinct climatic zones, each characterized by its unique seasonal patterns and an average temperature hovering around 28°C.

Five species of vulture are found in Côte d'Ivoire: the Hooded Vulture, the White‐backed Vulture ( *Gyps africanus* Salvadori, 1865), the Lappet‐faced Vulture (*Torgos tracheliotos* Forster, 1791), the White‐headed Vulture, and the Palm‐nut Vulture ( *Gypohierax angolensis* Gmelin, 1788). Aside from the Palm‐nut Vulture, all the other species are threatened according to the IUCN Red List and are primarily confined to the CNP (Salewski 2000; IUCN 2024). The decline in vulture populations in Côte d'Ivoire is attributed to several complex factors, including their use in traditional medicine, a threat that is often overlooked. Under Law No. 94‐442 of 16 August 1994, amending Law No. 65‐255 of 4 August 1965 on the protection of wildlife and the regulation of hunting (DFRC 2003), certain wild animal species in Côte d'Ivoire are afforded full protection. The capture, hunting and trade of these animals, including their young and eggs, are strictly prohibited. Only individuals holding scientific permits are permitted to capture them, and then solely under the conditions and using the methods outlined in their authorization. This protection extends unequivocally to all vulture species.

## 2 Methods

### 2.1 Market Surveys

**FIGURE 1:**
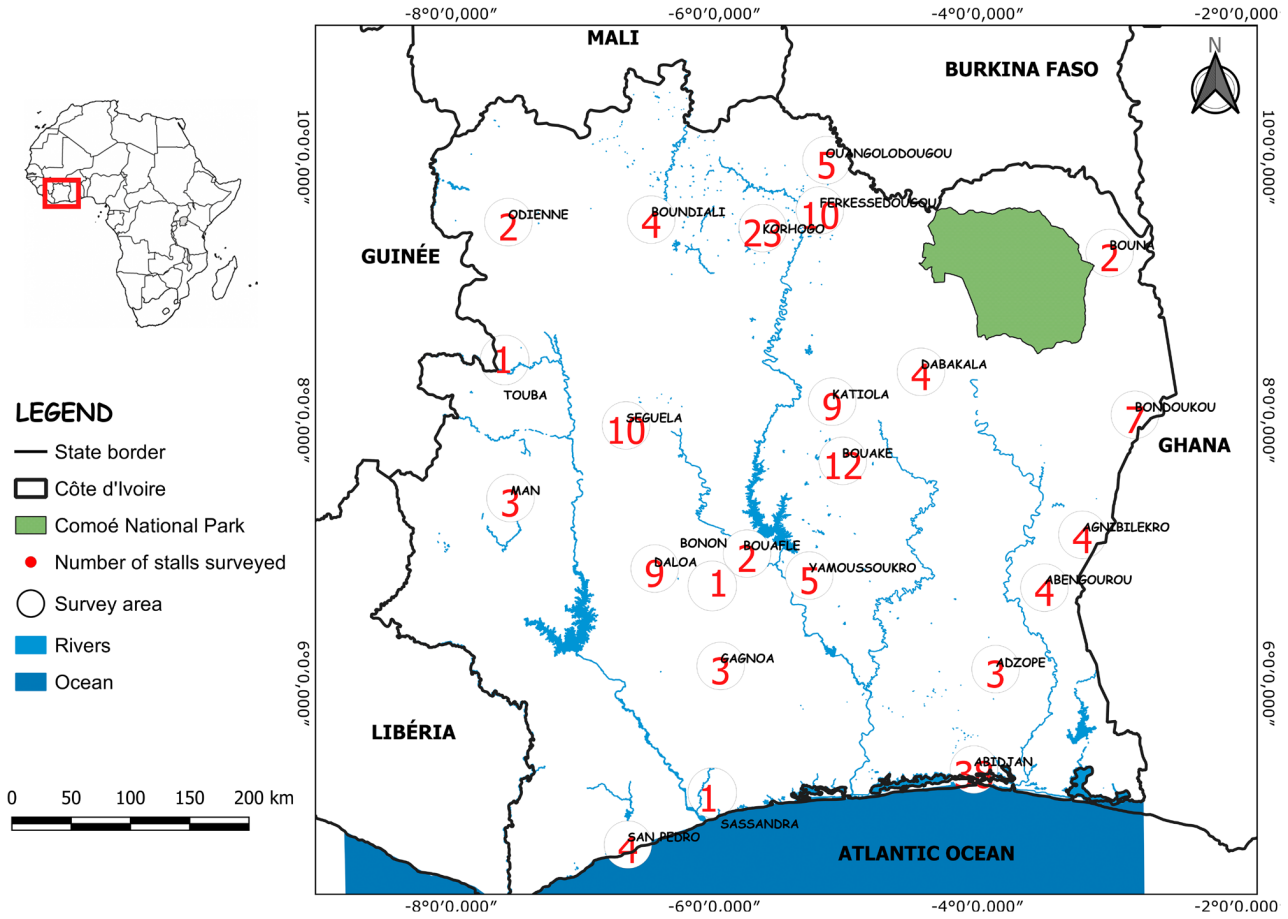
Cities and the number of stalls visited by city throughout the study of Côte d'Ivoire.

This study encompassed an extensive examination spanning all regions of Côte d'Ivoire, encompassing 24 cities from October 2021 to April 2022. Given the national scale of the survey, it was imperative to encompass a diverse array of towns across the country (Figure 1).

Prior to commencement, towns in each region had been preliminarily selected for inclusion in the survey. However, as the study progressed, adjustments were made based on insights gleaned from initial town selections within each region. Various regions, notable for their ethnic heterogeneity and attitudes toward vultures, which could potentially vary, including their potential usage in traditional medicine, were thoroughly surveys. Within each visited city, efforts were directed toward markets where vendors displayed dead animals or animal parts (Saidu and Buij 2013). A meticulous exploration was undertaken across 30 markets and 166 stalls. Each stallholder encountered was systematically invited to participate in the ethno‐ornithological survey and to respond to questions upon providing their consent (see Appendix S1). Adherence to ethical standards, as outlined by the British Sociological Association (BSA 2017), was a priority, ensuring that informed verbal consent was obtained prior to each interview. Ethical approval was obtained from the high scientific committee for ethical evaluation of the Université Nangui ABROGOUA (UNA) in Abidjan, Côte d'Ivoire (reference: N°266/MESRS/UNA/UFR‐SN) prior to the start of the data collection phase of this study. Of the 166 stallholders approached, 154 agreed to fully participate in the process, which comprised semi‐structured individual interviews and informal discussions. These interviews were conducted in French or Malinké (commonly referred to as Dioula) between 9:00 AM and 4:00 PM, with the interview venue accommodating the preference of the respondent, often beside their stalls. The surveys were conducted by the same individual (i.e., the corresponding author), with the assistance of locally selected aides in each of the regions visited. These aides acted as intermediaries, serving as ice‐breakers and interpreter‐guides when required. This approach fostered trust with our interlocutors, thereby ensuring the collection of accurate, comparable and representative information. This proximity facilitated direct observation, enabling the identification of all exhibited animals or animal parts. Although the primary focus centered on vultures and their components, data encompassed all discernible species present on the visited stalls.

With the proprietors' approval, photographs of the stalls were captured to aid in species identification (Whiting, Williams, and Hibbitts 2013). Species identifications were conducted with the assistance of field guides, notably those pertaining to West African birds (Borrow and Demey 2001). No personal information regarding respondents was collected, and the compiled database was meticulously anonymized.

### 2.2 Data Analyses

The data underwent conversion into responses of frequencies, followed by an analysis of variance using a Generalized Linear Model (GLM). We used this model to determine the relationship between the response variable and the following potential explanatory variables: origin of the sold vultures (Nominal qualitative variable indicating whether the sold vultures originated from Côte d'Ivoire or foreign countries), trade actors (Nominal qualitative variable identifying the various suppliers, traders and associated clientele), frequency of supply and sale of vultures on the stalls (Quantitative variable measuring the number of times traders receive and sell vultures or their parts), durability of stalls selling vultures (ordinal qualitative variable evaluating the number of years these stalls have been in operation), price of sold vultures on the market (quantitative variable measuring the price of vultures or their parts sold on the stalls) and region was used as an explanatory variable. We have specified a Poisson distribution. This analysis was tailored to achieve several objectives: (i) reflecting the diverse sources of vulture supply across different regions of Côte d'Ivoire, (ii) discerning the provenance of vultures sold within each visited region, (iii) evaluating the frequency of vulture trade or replenishment relative to the organs sold and the geographical regions and (iv) assessing of the quantity of vultures sold, alongside the frequency of trade or restocking of vultures based on the organs traded and regional characteristics. Furthermore, the impact of the number of stalls and the life span of stall within the visited regions on vulture availability and the availability of vulture parts was investigated. The analyses were conducted using the R Software (R Core Team 2021).

Given the inherent challenge of comprehensively cataloging all observed animal species within such a study, various methods were used to estimate the total species richness from a representative sample (Chiarucci et al. 2003). This species richness metric is correlated with the total count of individuals across all recorded species (Williams 2003), with species exhibiting higher abundance on the stalls being more likely to be recorded (Buzas and Hayek 1998). Incidence‐based species richness estimators, computed using PAST version 4.3 software, were consequently utilized.

A correspondence factorial analysis was carried out on a contingency matrix or frequency table between the regions visited and the causes mentioned (Lebreton et al. 1988), with the aim of determining the existence of a possible relationship between the causes of the high price of vultures marketed in the different regions of Côte d'Ivoire. It is based on a Chi‐square metric and considers symmetrically the rows and columns of the table.

Considering both species richness and the incidence (or abundance) of vulture species traded in the market, the Shannon diversity index was computed (Magurran 2013; Whiting, Williams, and Hibbitts 2013). This index ranges between 1.3 and 3.5, with lower values indicating fewer species and a higher dominance of a few species within the sample (Margalef 1972). Conversely, the index value increases with greater species richness (Colwell 2009). Piélou's equitability index (*J*) was employed to further elucidate this Shannon index.

The data used from the 166 stalls with vultures exhibited were used to run a simple linear regression model in which the dependent variable is the number of vultures sold per year and the independent variable is the number of stalls (Kutner et al. 2005; Wooldridge 2013).

## 3 Results

### 3.1 Characteristic of Respondents

The study encompassed a diverse range of respondents, spanning from individuals aged 21 years to those surpassing 60 years (Table 1). The age cohort of 51–60 years emerged as the most prevalent, constituting 39.39% of the participants, closely followed by the 41–50 years age group at 34.09%. Individuals aged 31–40 years comprised 9.09% of the cohort, whereas those aged 60 years and above constituted a smaller proportion, at merely 4.55%. The youngest subset, aged between 21 and 30 years, represented a mere 1.52% of the total respondents.

**TABLE 1:** Distribution of respondents' religious affiliation across gender and age groups.

Sex	Age	Religion		Total (*n* = 128)
		Animist (*n* = 20)	Muslim (*n* = 108)	
Male (*n* = 114)	21–30 (*n* = 2)	0	2 (1.43%)	2 (1.43%)
	31–40 (*n* = 12)	5 (3.57%)	7 (5%)	12 (8.57%)
	41–50 (*n* = 45)	6 (4.29%)	39 (27.86%)	45 (32.14%)
	51–60 (*n* = 50)	5 (3.57%)	45 (32.14%)	50 (35.71%)
	≥ 61 (*n* = 5)	1 (0.71%)	4 (2.86%)	5 (3.57%)
Female (*n* = 14)	21–30 (*n* = 1)	0	1 (0.71%)	1 (0.71%)
	31–40 (*n* = 2)	0	2 (1.43%)	2 (1.43%)
	41–50 (*n* = 8)	1 (0.71%)	7 (5%)	8 (5.71%)
	51–60 (*n* = 3)	2 (1.43%)	1 (0.71%)	3 (2.14%)

Most respondents were male (88.64%), with a notable proportion holding foreign nationalities (Table 2). Predominantly, the Haoussas from Niger formed the largest demographic (82.56%), with a solitary representation from the Ghanaian Akan (0.76%). Among those of Ivorian nationality, the Sénoufos ethnic group constituted the largest segment (13.64%), trailed by Djiminis (2.28%) and Malinkés (0.76%) (Refer Table 2).

**TABLE 2:** Ethnicity distribution among respondents classified by gender and age.

Sex	Age	Ethnic					Total (*n* = 132)
		Sénoufo (*n* = 18)	Djimini (*n* = 3)	Malinké (*n* = 1)	Haoussa (*n* = 109)	Akan (*n* = 1)	
Male (*n* = 117)	21–30 (*n* = 2)	0	0	1 (0.76%)	1 (0.76%)	0	2 (1.52%)
	31–40 (*n* = 12)	3 (2.27%)	0	0	8 (6.06%)	1 (0.76)	12 (9.09%)
	41–50 (*n* = 45)	7 (5.30%)	0	0	38 (28.76%)	0	45 (34.09%)
	51–60 (*n* = 52)	6 (4.55%)	1 (0.76%)	0	45 (34.09%)	0	52 (39.39%)
	≥ 61 (*n* = 6)	0	1 (0.76%)	0	5 (3.79%)	0	6 (4.55%)
Female (*n* = 15)	21–30 (*n* = 1)	0	0	0	1 (0.76%)	0	1 (0.76%)
	31–40 (*n* = 2)	0	0	0	2 (1.52%)	0	2 (1.52%)
	41–50 (*n* = 9)	2 (1.52%)	1 (0.76%)	0	6 (4.55%)	0	9 (6.82%)
	51–60 (*n* = 3)	0	0	0	3 (2.27%)	0	3 (2.27%)

Regarding religious affiliation, a substantial proportion of the respondents were Muslims (77.14%, *n* = 108), while a smaller subset were Animists (14.29%, *n* = 20) (Table 1).

Significant biases were observed across multiple demographic variables, notably favoring men (*χ* ^2^1 = 90.754, *p* < 0.001), particularly those of foreign nationality, specifically the Haoussas (*χ* ^2^2 = 13.036, *p* < 0.05), adherents of Islam (*χ* ^2^3 = 84.004, *p* < 0.001) and individuals belonging to the older age cohorts (*χ* ^2^4 = 17.012, *p* < 0.001).

### 3.2 Diversity of Vultures or Parts of Vultures Found on the Market Stalls Visited

**FIGURE 2:**
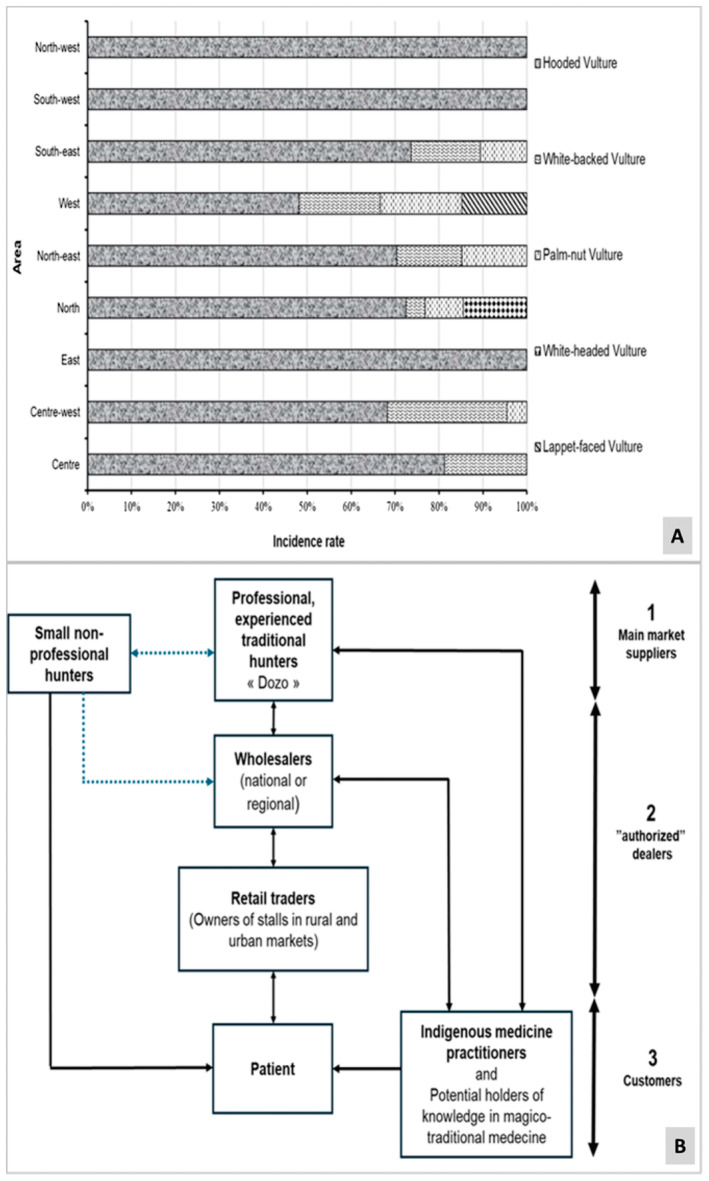
Trade patterns and market dynamics of vulture species in Côte d'Ivoire. (A) Variation in the incidence rate of vulture species sold at market stalls; (B) Marketing circuit of vultures and their parts in Côte d'Ivoire.

During our survey of market stalls, we identified five distinct species of vultures. These comprised the Hooded Vulture ( *Necrosyrtes monachus* ), the White‐backed Vulture ( *Gyps africanus* ), the Palm‐nut Vulture ( *Gypohierax angolensis* ), the White‐headed Vulture ( *Trigonoceps occipitalis* ) and the Lapped‐faced Vulture (*Torgos tracheliotos*). The Hooded Vulture emerged as the predominant species, accounting for 71.10% of sightings, followed by the White‐backed Vulture at 12.80%, the Palm‐nut Vulture at 9.48%, the White‐headed Vulture at 4.74% and the Lapped‐faced Vulture at 1.90%. Remarkably, the Hooded Vulture exhibited consistent prevalence across all surveyed regions, with sole representation in some areas such as the South‐West, East and South‐West. Notably, simultaneous presence of all five species (Hooded Vulture, White‐backed Vulture, Palm‐nut Vulture, White‐headed Vulture and Lapped‐faced Vulture) was never recorded on any single stall. Statistical analysis revealed a significant regional variability in species incidence rates (GLM: df = 7; *p* < 0.001; Figure 2A).

The Shannon diversity index ranged from 0 to 0.94 (Table 3), indicating a decrease in overall vulture species richness in the stalls studied. Conversely, regions recording an index value of 0 signify the exclusive presence of a solitary vulture species. Remarkably, the Piélou equitability index shows high values, attributed to the prevalence of 0 values in the Shannon index.

**TABLE 3:** Variability in vultures species sold within Côte d'Ivoire Marketplaces.

	Visited areas								
	Central	Central west	East	North	North‐East	West	South‐East	South‐West	North‐West
Specific richness	2	3	1	4	3	4	3	1	1
Incident numbers	16	22	9	69	27	27	38	3	4
Shannon diversity index (H′)	0.48	0.76	0	0.86	0.81	1.26	0.75	0	0
Piélou equitability index (J)	0.70	0.69	—	0.62	0.74	0.91	0.69	—	—

### 3.3 Supply of Vultures to Market Stalls: Origin, Actors and Circuit

The vulture trade in Côte d'Ivoire is intricately woven into a well‐established hunting and supply chain, involving key players across three distinct categories: primary market suppliers, authorized dealers and consumers. Among these, primary suppliers comprise seasoned traditional hunters, known locally as “Dozos,” and occasional providers who engage in hunting upon commission. According to the data collected, around 70% of the vultures marketed come from Dozos hunters. Notably, certain traditional hunters, accounted for approximately 25% of this group, are revered in their communities for possessing healing abilities, motivating their hunts to procure medicinal supplies for their patients.

Authorized dealers operate either as wholesalers or retailers. Wholesalers primarily cater to market stalls, operating on both national and regional scales. On average, each wholesaler managed 4–10 stalls, enabling them to extend their reach to several regions or the whole country. Conversely, retail traders exclusively manage stalls in local markets, receiving deliveries and directly vending to consumers.

The end consumers consist of traditional practitioners (65%), often referred to as fetishists, or individuals (35%) seeking knowledge in magico‐traditional medicine. These consumers acquire vultures or vulture parts for various purposes, including addressing health issues of biological, mystical or mental origins.

The marketing circuit in Côte d'Ivoire begins with a demand from a magico‐traditional practitioner, commonly known as a fetishist, who, to address diverse health concerns, solicits vulture or vulture parts from patients (Figure 2B). These patients may then procure the sought‐after items from markets directly or through discreet channels, facilitated by occasional suppliers. Moreover, traditional practitioners occasionally supply animal parts from their own stock to patients requiring remedies involving vulture components.

**FIGURE 3:**
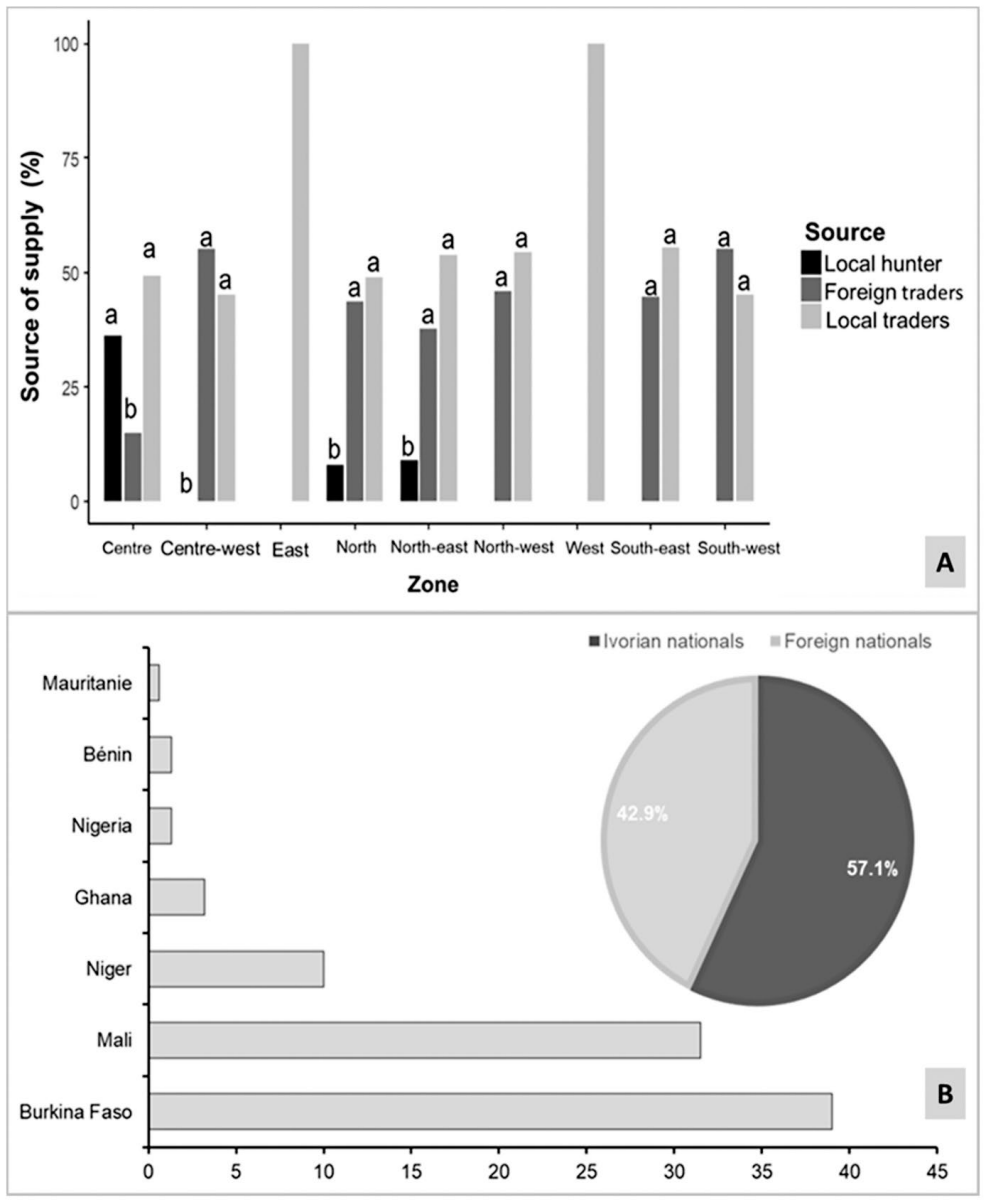
Trade and origin of vultures species in the Ivorian Magico‐traditional medicine market. (A) Provision of vultures and their parts by merchants across varied locales within Côte d'Ivoire; (B) The provenance of vultures contributing to the Ivorian traditional magico‐traditional medicine market as per insights from interviewed stall owners. Values with different letters (a and b) are significantly different at *p* = 0.05.

The traders interviewed conveyed that their vulture or vulture parts supply stemmed from either local or foreign wholesale traders, or alternatively from poachers (or traditional hunters). Among them, 6.90% indicated poachers as their source. Predominantly, wholesale traders (48.62%) and foreign traders (44.48%) were cited as the primary sources. Notably, a significant discrepancy in involvement among these various entities was discerned (GLM: df = 2, *p* < 0.001), alongside regional disparities (Figure 3A), wherein supply sources varied across regions.

In Côte d'Ivoire's lively markets, vulture sales are spread across a wide demographic range. Astonishingly, 57.10% of these raptors originate from Ivorian nationals, comprising both traditional professional hunters and wholesale traders, while 42.90% hail from individuals of foreign descent. Delving deeper, it becomes evident that 68.80% of vultures flooding the market are locally sourced by wholesale traders, with an additional 10.40% stemming from indigenous traditional professional hunters. Surveying the origins of imported vultures, a substantial 42.55% are reported to journey from Burkina Faso, closely followed by 34.75% from Mali. Other nations contributing to the Ivorian vulture market include Ghana (7.80%), Niger (7.09%), Nigeria (1.42%), Benin (1.42%) and Mauritania (1.42%). Intriguingly, statistical analysis using the examination of variance within the framework of the General Linear Model reveals no significant difference between vultures originating from foreign nationals and those originating from Ivorian nationals. (df = 1; *p* > 0.05; Figure 3B).

### 3.4 Frequency of the Availability and Sale of Vultures and Vulture Parts at Market Stalls

Through direct observations conducted at market stalls, we were able to discern the assortment of vulture parts prominently exhibited. Our findings reveal that the head (43.44%) emerged as the most prevalent item, succeeded by feathers (27.05%), whole vultures (14.75%), legs (9.02%) and bones (5.74%). According to some traders, live vultures and eggs could also be supplied on a made‐to‐order basis.

The data illustrate a periodic restocking of vultures, or their components observed at these stalls, occurring at intervals spanning 1–4 months. However, the fulfillment of specific orders, such as those for vulture eggs, may prolong this timeline depending on demand. Notably, a substantial variance in restocking frequency was noted in accordance with citations (GLM: df = 4 *p* < 0.001).

**FIGURE 4:**
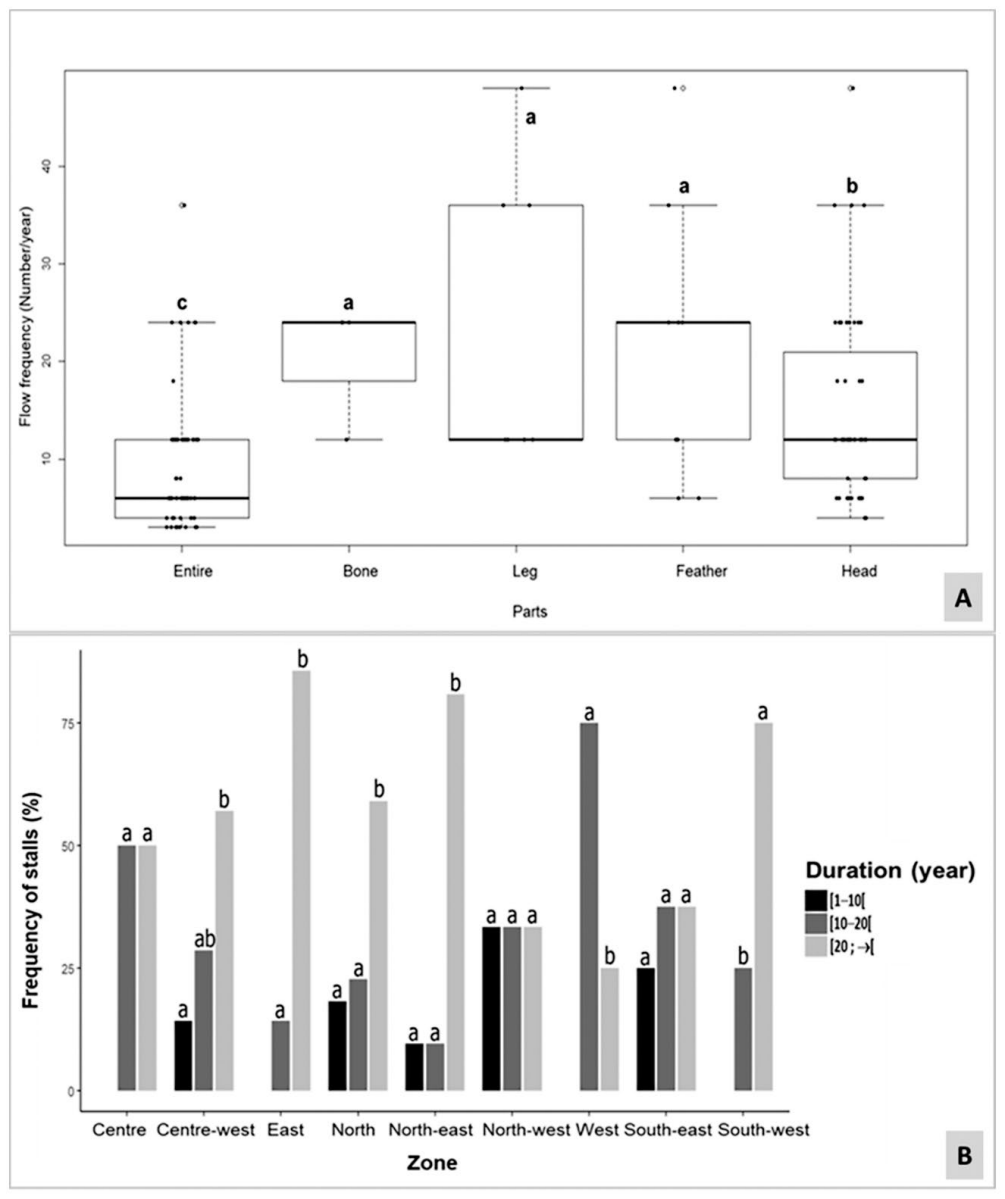
Analysis of vulture species trade dynamics: turnover and temporal extent across regions. (A) Mean annual turnover of vultures and their parts per interviewed trader; B: Temporal Extent of Stalls Across Regions. Values with different letters (a, b, and c) are significantly different at *p* = 0.05.

Furthermore, our analysis unveils that the mean annual restocking frequency per trader diverges based on the vulture component (GLM: df = 8 *p* < 0.001; Figure 4A). Specifically, the highest frequency is recorded for legs (24 ± 15.49 legs), succeeded by bones (21 ± 6 bones) and feathers (21.33 ± 14.10 feathers). Conversely, the frequency is moderately elevated for heads (14.77 ± 9.19 heads) and whole vultures (9 ± 6.73 whole vultures).

### 3.5 Stall Life Span in the Trade of Vultures and Their Parts

The resilience and continuity of stalls engaged in the trade of vultures and vulture derivatives exhibit notable variation. Across all surveyed regions, 14.86% of stalls had a tenure of less than a decade, 29.05% operated for fewer than 20 years and a majority, 56.08%, endured beyond the two‐decade mark. Analysis employing General Linear Modeling (GLM) underscores a statistically significant variance in stall counts relative to their operational longevity (df = 2; *p* < 0.001). Moreover, pronounced disparities emerge in stall numbers across distinct regions (df = 8; *p* < 0.001). Notably, both temporal longevity and geographical locale exert a significant influence on stall abundance (df = 13; *p* < 0.001; Figure 4B).

**FIGURE 5:**
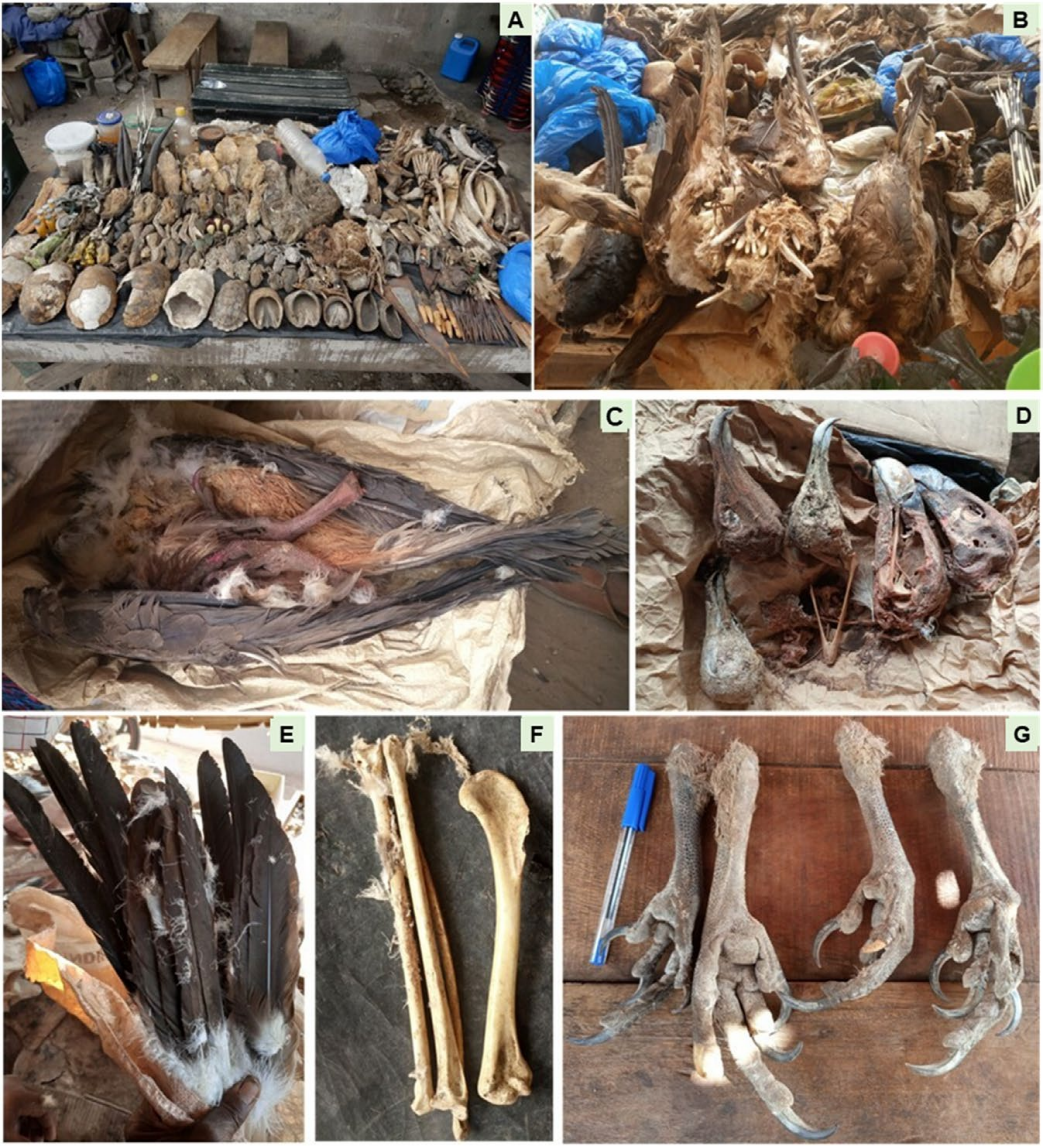
Stalls showcasing diverse fauna and vulture specimens, alongside their respective groups, within a regional marketplace; and Vulture and their parts found in some local markets in Côte d'Ivoire. (A) Denotes booths exhibiting a spectrum of animal species, while B denotes booths featuring vultures and their kin in the local market setting. (C) Entire Hooded Vulture ( *Necrosyrtes monachus* ); (D) Heads of Hooded Vulture ( *Necrosyrtes monachus* ), White‐backed Vulture ( *Gyps africanus* ) and Lapped‐faced Vulture (*Torgos tracheliotos*); (E) Feathers of a Palm‐nut Vulture; (F) Some bones of vulture; (G) Legs of different vulture species.

The most enduring stalls were predominantly observed in central‐western, eastern, northern, north‐eastern, western and south‐western regions, whereas nascent establishments were predominantly clustered in central, north‐eastern and south‐eastern locales. However, instances of recent stall inception were also noted in regions hosting long‐standing establishments. Figure 5 shows some stalls showcasing diverse fauna and vulture specimens.

### 3.6 Selling Prices of Vultures and Their Parts in the Visited Markets

The prices at which vultures and their various components are sold exhibit notable variation across different regions, while demonstrating relative consistency within specific locales (Generalized Linear Model: df = 8; *p* < 0.001; Table 4). Furthermore, these selling prices exhibit discernible disparities among different vulture components (GLM: df = 4; *p* < 0.001). In regions where expenses are particularly steep, the price for an entire vulture can reach up to US$ 160. However, merchants unanimously agree that the practice of skinning and retailing individual vulture parts proves more financially lucrative than selling the whole bird outright. Indeed, prices can soar to US$ 75 for a vulture's head and US$ 14 for a single leg. Vulture bones, contingent upon their size and the specific limb from which they originate, fetch prices of US$ 8 or more each, while feathers command a rate of US$ 2 per unit.

**TABLE 4:** Average retail prices of vultures or their components at market stalls across surveyed regions.

	Mean ± SE (USD)				
	Entire	Head	Bone	Leg	Feather
North‐east	159.59^a^ ± 35.64	49.79^c^ ± 9.67	5.23^b^ ± 1.64	10.77^a^ ± 3.66	1.65^ab^ ± 0
Center	133.90^b^ ± 26.42	57.11^b^ ± 16.68	3.30^c^ ± 0	11.83^a^ ± 5.50	1.63^ab^ ± 0.12
West	126.64^bc^ ± 8.53	74.33^a^ ± 12.79	8.26^a^ ± 0	13.76^a^ ± 4.26	1.38^bcd^ ± 0.43
South‐west	103.24^cde^ ± 25.06	50.01^bc^ ± 20.32	—	8.17^b^ ± 0.39	0.92^d^ ± 0.27
Centre‐west	103.41^cd^ ± 27.34	46.35^c^ ± 14.68	6.30^ab^ ± 2.12	11.02^a^ ± 6.87	1.30^cd^ ± 0.48
South‐east	101.64 ^cde^ ± 31.50	44.17^c^ ± 12.71	—	8.83^ab^ ± 3.67	1.82^a^ ± 0.61
North	94.89^de^ ± 13.21	41.46^c^ ± 5.60	4.21^c^ ± 0.71	8.19^b^ ± 1.16	1.50^bc^ ± 0.36
East	89.93 ^de^ ± 8.70	36.71^c^ ± 4.35	—	9.36^ab^ ± 1.65	1.56^abc^ ± 0.27
North‐west	82.59^e^ ± 0	48.86^c^ ± 2.33	—	7.98^b^ ± 0.93	1.62^ab^ ± 0.17
Df	8				
*p* value >| t |	< 0.001***				

*Note:* Values with different letters (a, b, c, d, and e) are significantly different at *p* = 0.05.

On a national scale, however, the average price for an intact vulture (Figure 5) stands at US$ 110 ± 0.81. The head garners an average price of US$ 46 ± 1.15, a leg fetches US$ 9 ± 1.15, bones are valued at US$ 5 ± 1.82 each and feathers are priced at US$ 1.51 ± 1.15.

### 3.7 Changes in the Selling Prices of Vultures and Their Components Over Time

Respondents were unanimous in observing a steady escalation in the price of wild animals in general, and vultures in particular, over the last decades. The underlying factors driving this upward trend varied across different regions of the country (*χ* ^2^ = 784.95; df = 24; *p* < 0.001). According to respondents, the reasons for this sustained surge encompassed a spectrum of issues, from the dwindling numbers and potential extinction of vulture species to escalations in wholesale prices within the market. Stakeholders in the trade also attributed the increase to augmented customs duties.

In the northern reaches of the country, 71.14% (*n* = 28) of respondents cited vulture rarity, while 29.86% (*n* = 11) pointed to escalating wholesale prices as rationale for the continuous price hikes. In the North‐East, respondents attributed the phenomenon to species extinction (50.32%; *n* = 13) and exorbitant wholesale prices (49.68%; *n* = 12).

In the South‐East, 46% (*n* = 7) of respondents highlighted the rise in wholesale prices, whereas 54% (*n* = 9) underscored the scarcity of vultures in their natural habitats. Notably, in certain regions, an unexpected factor was the increase in customs duties, particularly prevalent in the south‐west and Central‐west. Conversely, the Central, Eastern, Western and North‐Western regions attributed the price hikes solely to vulture rarity.

**FIGURE 6:**
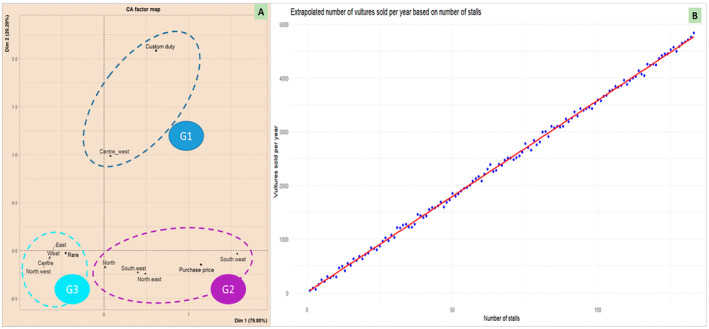
Market dynamics and trade estimates of vultures species in Côte d'Ivoire. (A) Factors contributing to the persistent escalation in the market valuation of vultures and their components across diverse regions explored in Côte d'Ivoire; (B) Extrapolated number of vultures sold per year based on number of stalls.

In summary, a correlation analysis between regions visited and the cited causes for the continual escalation in vulture sale prices revealed three distinct clusters (Figure 6A). The central‐western region constituted the first group, identifying customs duties as the primary driver of the price surge. The second group comprised the North, South‐East, North‐East and South‐West regions, where the price hikes were attributed to sustained increases in wholesale prices. Lastly, the third group, encompassing the West, North‐West and Central regions, ascribed the phenomenon to vulture scarcity or extinction.

### 3.8 Impact of the Trade and Use of Vultures in Magico‐Traditional Medicine on Population Decline

During this study, 166 market stalls were observed and documented. Based on the count of vulture heads on display, which represented whole individuals, it was found that at least three vultures were observed in 133 of these stalls (roughly 80%). Furthermore, the stall owners reported selling at least three vulture heads per month, equating to around 36 vultures per stall annually. Extrapolating these figures across the 133 vulture stalls suggests a total of approximately 4, 839 vultures sold each year nationwide. Although this estimate is indicative, it is deeply concerning, revealing (i) a very strong correlation between the number of stalls and the number of vultures sold annually (*r* = 0.99; *p* < 0.0001; Figure 6B), and (ii) a regional supply network supporting this high demand of vulture parts. It is also important to note that stall owners from the same market, town or region often purchase from one another based on the demand and availability of vultures in their respective stalls.

## 4 Discussion

This study has yielded a comprehensive evaluation of the vulture market dimensions in Côte d'Ivoire, along with identifying the diverse stakeholders engaged across its various stages. Furthermore, it facilitated the delineation of the entire trajectory from vulture hunting to their commercialization throughout the country. However, beyond the nationalities of the main players in this trade, it is essential to emphasize the deep‐rootedness in the collective consciousness of several West African peoples of the belief‐based use or their components in traditional magical medicine. Predominantly, traders surveyed hail from Niger, particularly from the Hausa ethnic group, as noted by Saidu and Buij (2013). This ethnic group is presumed to possess expertise or traditional medicinal knowledge employing animals and their parts in crafting remedies. These traders exhibit profound insight into the motivations driving the acquisition of vultures or their parts, indicative of their advanced proficiency in this specialized domain. Such considerable expertise affords the Hausa ethnic group significant leverage in dominating the trade of animals and their derivatives at local and regional levels.

The hunting and marketing circuit for vultures is firmly established, facilitated by a regional network that expedites supply, sometimes within a month. The expeditious acquisition of supplies by circuit participants may be attributed to the proximity of hunting grounds or the influence of a hunting and distribution network likely entangled with corruption. Within Côte d'Ivoire, Comoé National Park is suspected to serve as a primary sanctuary for vultures, thus constituting a pivotal supply hub for markets across the subregion. Furthermore, traders justify escalating prices of vultures and their parts on the market by citing increased customs duties. However, a customs duty is intended to be levied on legitimate trade, not on the illicit trafficking of animals with well‐defined conservation statuses, both domestically and internationally.

Scrutiny of the ramifications of the vulture trade and its impact on the sustainable preservation of these avian predators exposes several disquieting facets. As delineated by Daboné et al. (2023), the substantial profits derived from the vulture trade pose a significant impediment to their conservation efforts, particularly as this trade flourishes within impoverished socioeconomic milieus with complete impunity. The rapid turnover of fresh supplies and replenishments of vulture stocks in Côte d'Ivoire is striking, often being replenished in under 4 weeks, indicative of fervent domestic demand (Awoyemi 2014). Such fervor is liable to foment various forms of persecution against these raptors, thereby imperiling their regional biodiversity.

Prior investigations (Ogada and Buij 2011; Buij et al. 2016; Daboné et al. 2022), have underscored the lucrative allure of the vulture parts market, incentivizing traders to augment their stockpiles. Thiollay (2006) similarly observed the extensive utilization of vultures for medicinal and ceremonial purposes, precipitating the widespread peddling of their body parts in local markets. Analogous trends are discernible in other countries, such as Benin (Adjakpa, Tchabi, and Ogouvide 2002), Mozambique (Parker 2004), South Africa (McKean et al. 2013) and Nigeria (Williams et al. 2014; Williams et al. 2021) where the illicit trade in wildlife in general poses a significant hazard to species viability due to unsustainable hunting.

In West Africa, the demand for vultures in traditional medicinal practices has engendered various forms of persecution, encompassing poisoning and illicit hunting (Ogada, Botha, and Shaw 2016b; Botha et al. 2017; Margalida et al. 2008; Margalida et al. 2019; Henriques et al. 2020). Surveillance of vulture parts in markets evinces a preponderance of limbs, skeletal remains and plumage, indicative of a predilection for these elements in Ivorian traditional customs. This recurrent theme is buttressed by annual sales data, which underscore the persistent pressures exerted on these raptors in the West African domain, particularly within Côte d'Ivoire.

The preference for the anatomical features of vultures, such as legs, feathers and bones, is justified in part by their belief‐based use in healing rituals, divination practices and magical rites, as numerous studies attest (Owolabi, Odewumi, and Agbelusi 2020; Saidu and Buij 2013; Mullié et al. 2017). Within Côte d'Ivoire, these sought‐after parts are coveted for their perceived qualities of longevity, safeguarding and mystical potency (Asso, Koné, and Salewski 2024). In essence, these revelations underscore the intricate dilemmas confronting vulture conservation amidst a backdrop of escalating demands for their anatomical components for traditional cultural and medicinal applications.

The Hooded Vulture emerges as the most extensively traded species, albeit sightings of other species are sparse and typically in limited quantities. This disparity may be attributed not solely to the disappearance of larger vulture species but also to the foraging behavior of Hooded Vultures, commonly encountered in slaughterhouses and refuse sites within villages and cities. This makes this species very vulnerable to poaching, as indicated by several authors (Nikolaus 2001, 2011; Ogada and Buij 2011; Sodeinde and Soewu 1999). In Nigeria, specimens of Hooded Vultures are widely sold through traditional medicine traders (Sodeinde and Soewu 1999; Williams et al. 2021). Similarly, in Burkina Faso, the Hooded Vulture finds widespread application in traditional medicine (Ducatez et al. 2007; Saidu and Buij 2013). Such a pronounced focus on Hooded Vultures underscores the challenges faced by actors in the vulture trade in sourcing other species. It could also be a sign of the increasing scarcity of other species and, above all, contribute to the rapid extinction of the Hooded Vultures. Ultimately, it appears that for actors of this trade, one vulture is interchangeable with another. Specific distinctions between species are not discerned, thus the species most abundant or easiest to poach is most likely to be found market stalls.

To address the urgent threat of vulture extinction in Côte d'Ivoire and the broader West African subregion, it is essential to implement integrated measures at both national and regional levels. These measures must encompass a range of initiatives, balancing the conservation of these raptors with the needs of local communities, particularly concerning traditional medicine practices. Initially, organizing national and regional meetings among stakeholders, including policymakers, scientists, practitioners, NGOs and civil society, is crucial for exchanging and harmonizing ideas. The findings of this study can partially contribute to these discussions, having identified key actors involved in the hunting and trading of vultures. However, a comprehensive mapping of national and regional stakeholders is necessary before these meetings can take place. In addition, conducting awareness and education campaigns to inform communities and those engaged in the vulture trade about the ecological significance of these organisms and the ramification of their decline is vital. These campaigns should highlight the environmental impact and the consequences for local livelihoods. Mainstreaming vulture conservation into school curricula and environmental education programs is also important. At the same time, it is crucial to engage in close collaboration with (i) stakeholders in the trade of vulture parts, particularly traditional healers, and (ii) local communities to conduct awareness campaigns aimed at preventing the use of poisoned bait. Identifying and promoting alternatives to vulture parts, while raising awareness of these substitutes, is crucial. This endeavor could be supported by bringing together traditional healers and practitioners of traditional medicine in partnership with NGOs and relevant government ministries responsible for environmental protection.

A focused protection policy for the Comoé National Park in Côte d'Ivoire, currently the last significant refuge for these raptors, must be implemented. This policy should include specific measures for monitoring, surveillance and protection of vulture populations, necessitating the mapping of their movements within and outside the park to develop a management policy informed by scientific research on vultures. On a political level, it is advisable to strengthen regulation and surveillance by strictly enforcing laws prohibiting the hunting and illegal trade of vultures. This effort should be supported by systems to detect illicit activities. By implementing these comprehensive measures, the conservation of vultures in Côte d'Ivoire and the broader West African subregion can be effectively addressed, ensuring the survival of these vital raptors while respecting the needs and practices of local communities. All these actions are in line with the Action Plan for the Conservation of Vultures in West Africa 2023–2043 and the strategic framework of the Multi‐species Action Plan for Vultures developed under the aegis of the Convention on Migratory Species (CMS) (Botha et al. 2017).

## 5 Conclusion

This study forms an integral component of a comprehensive evaluation concerning the commerce of vultures and/or their components within Côte d'Ivoire, accompanied by an examination of its ramifications on a regional scale. The findings underscore the lucrative nature of animal trade in general, with a particular emphasis on the trade in vultures. This trade predominantly falls under the purview of male participants and spans the entirety of the nation, facilitated by a sophisticated hunting and distribution network catering to both national and subregional markets. Such activity is sustained by an ongoing demand, leading to a consistent escalation in prices and mounting pressure on vulture populations, especially within their remaining natural habitats such as parks and reserves. The sustainable conservation of these raptors presents a multifaceted challenge, exacerbated by the presence of commercial stalls some established decades ago, others more recently which pervade all marketplaces. The substantial profitability of this enterprise perpetuates its existence, even amidst precarious socioeconomic conditions, often with complete impunity. Considering these revelations, it becomes imperative to contemplate concerted action at the subregional level, geared toward safeguarding vulture populations in Côte d'Ivoire and the broader West African subregion. This endeavor may entail bolstering legislative frameworks and implementing measures to combat the illicit vulture trade, along with awareness‐raising campaigns on the services and role of vultures in the ecosystem and education to cultivate a harmonious coexistence between human activities and biodiversity conservation. Furthermore, the establishment of monitoring and research initiatives is indispensable for a more profound understanding of vulture population dynamics and ecological requisites, thereby guiding conservation strategies toward efficacious and suitable long‐term solutions.

## Author Contributions

**Asso Armel Asso:** investigation (equal), methodology (equal), writing – review and editing (equal). **Volker Salewski:** conceptualization (equal), supervision (equal), validation (equal), visualization (equal), writing – review and editing (equal). **N'golo Abdoulaye Koné:** conceptualization (equal), methodology (equal), project administration (equal), supervision (equal), validation (equal), visualization (equal), writing – original draft (equal), writing – review and editing (equal).

## Ethics Statement

This study complied with the ethical guidelines of the British Sociological Association. Informed consent was obtained verbally from each survey participant prior to the interview, and participants were informed of their right to participate voluntarily or to refuse.

## Conflicts of Interest

The authors declare no conflicts of interest.

## Supporting information


Data S1

## Data Availability

For security reasons, sensitive data regarding the precise locations and targeted species have been kept confidential to prevent misuse, such as for poaching or illegal trade. However, these details can be made available upon written request to the authors at: 04 BP 1036 Abidjan 04.
