# Supplementary material for: Unveiling the Threat to Vulture Diversity: A Comprehensive Ethno‐Ornithological Study Uncovers Regional Trade Effects in Côte d'Ivoire
Source: Ecol Evol. 2024 Dec 23;14(12):e70740. doi: 10.1002/ece3.70740 (PMC11666337; doi:10.1002/ece3.70740)
Supplement: Supplementary file 1 — Data S1 [file ECE3-14-e70740-s001.pdf]

QUESTIONNAIRE D'ENQUÊTE SUR LES VAUTOURS DANS LES MARCHES

Fiche N° ....

Date :...

Localité : ....

Commune :......................................

Demande de consentement à participer à l'entretien d'enquête

Dans le cadre d'une étude qui vise à mieux comprendre les connaissances traditionnelles et l'étendue du trafic et du commerce des vautours et de leurs parties sur les marchés ruraux et urbains de Côte d'Ivoire, nous vous invitons à participer à un entretien. Cet entretien consiste en un questionnaire qui nous aidera à recueillir des informations essentielles pour notre recherche.

Votre participation est entièrement volontaire. Vous êtes libre de refuser de participer ou de vous retirer à tout moment sans avoir à fournir de justification, et sans que cela n'entraîne de conséquences négatives pour vous.

Les informations que vous fournirez seront traitées de manière strictement confidentielle et anonymisée. Elles ne seront utilisées qu'à des fins de recherche scientifiques et ne seront partagées qu'avec les membres de l'équipe de recherche. Aucun renseignement personnel permettant de vous identifier ne sera divulgué.

Nous espérons que vous accepterez de participer à cet entretien, qui est d'une grande importance pour la conservation des vautours et la préservation de l'écosystème en Côte d'Ivoire.

Si vous avez des questions ou des préoccupations concernant cette recherche, n'hésitez pas à nous le dire.

Veuillez indiquer votre consentement en signant ci-dessous.

Consentez-vous à participer à cet entretien ☐ Oui ☐ Non

Identification de l'interrogé

- **Âge :** ☐ **≤20** ☐ **21-30** ☐ **31-40** ☐ **41-50** ☐ **51-60** ☐ **≥61**
- **Sexe :** ☐ **M** ☐ **F**
- **Groupe ethnique : ....**
- **Profession :.. ....**
- **Religion : ....**
- **Niveau académique :..**

Niveau de connaissance des vautours par l'enquêté

1. Connaissez-vous les vautours ☐ Oui ☐ Non
2. Vendez-vous des vautours et/ou paries de vautours sur votre étal ? ☐Oui ☐Non
   Si oui pouvez-vous nous en montrer sur votre étal ?

3. Depuis combien de temps exercez-vous ce métier de vente d'animaux et de parties d'animaux ?..
4. Combien d'étals de ce genre y'a-t-il dans votre marché ?

....
....

5. Ces étals ont-ils été implantés au même moment ?

....
....
....

6. Depuis quand l'étal le plus ancien de votre marché a-t-il été établit ?

....
....

7. Quand est-ce que l'étal le plus récent de votre marché a-t-il été établit ?

....
....

8. Comment est-ce que vous vous approvisionnez en vautours ?
   ☐ Chasse moi-même ☐ Chasseurs locaux ☐ Chasseurs étrangers
   ☐ Vendeurs locaux ☐ Vendeurs étrangers
   Précisez si possible ....

9. Quelles sont les provenances des vautours que vous avez sur votre étal ?

☐ Locale

☐ Autres régions de la Côte d'Ivoire, précisez si possible ....

☐ Étranger, précisez les pays si possibles ....

10. Combien de types (espèces) de vautours avez-vous l'habitude de vendre ?

☐ 1 ☐ 2 ☐ 3 ☐ 4 ☐ 5 ☐ Plus ?

Caractéristiques de la clientèle

11. Qui sont vos clients ?

☐ Hommes ☐ Femmes ☐ Tradipraticiens

12. Savez-vous ce que vos clients font des vautours ou parties de vautours qu'ils achètent avec vous ? ☐ Oui ☐ Non

Si oui pouvez-vous citez quelques traitements dans lesquels les vautours ou parties de vautours sont utilisées ?...

13. À défaut de vautours est-ce vos clients ont souvent recours à d'autres animaux ou parties d'animaux pour leurs besoins respectifs ?

14. Qui fait les prescriptions à vos clients ? (☐ Vous-mêmes ? ☐ les Marabouts ? ☐ les guérisseurs ?, ☐ les féticheurs ? ☐ autres ? Précisez…………………………)

Vente des vautours : gain et circuit

15. Combien de vautours ou parties de vautours pouvez-vous vendre ?

☐ Par jour, précisez si possible ....

☐ Par semaine, précisez si possible ....

☐ Par an, précisez si possible ....

16. À quelle fréquence obtenez-vous de nouveaux stocks de vautours ?

☐ < 4 semaines

☐ 2 mois

☐ 3 mois

☐ > 4 mois

17. Qui est-ce qui vous fournit en vautours ou parties de vautours ?

……………………………………………………………………………………………..

……………………………………………………………………………………………..

18. Depuis combien de temps faites-vous ce commerce de vautours

☐ 1-10 ans ☐10-20 ans ☐ 20 ans et plus

19. Quels sont les différents prix pratiqués durant la vente de vautours et/ou de parties des vautours ?

**Libellé**	**Prix d'achat**	**Prix de vente**
Vautours entiers		
Tête		
Pattes		
Plumes		
Œufs		
Autres organes (précisez)		

20. Comment évoluent les prix de vautours entiers ou parties de vautours au cours de années ?

☐ Augmentation ☐Diminution ☐Constance

21. Est-ce que le type (espèce) de vautour influence le prix du vautour ou de ces parties ? Si oui, pourquoi ?...

………………………………………………………………………………………………

………………………………………………………………………………………………

………………………………………………………………………………………………

22. Quels sont les types (espèces) de vautours les plus demandés ou recherchés par vos clients ? ……………………………………………………………………………

………………………………………………………………………………………………

………………………………………………………………………………………………

23. À quelle période êtes-vous approvisionnée en vautours ?

☐ Saison sèche ☐ Saison pluvieuse ☐ Toute l'année

Précisez le mois..

Niveau de contrôle et de surveillance de la chasse illégale

24. Recevez-vous la visite des agents de l'état pour des contrôles ? si oui quel l'objet de ces visites ? Si oui qui fait ces contrôles et à quelle fréquence ?
25. Il est clair que vous vendez des animaux et parties d'animaux ; toutefois quelle est la conduite qu'ils tiennent pendant ces contrôles ? confiscation ? amendes ? peine de prisons ? autres, précisez s'il vous plait.

Si grande fréquence de contrôle :

Pourquoi avec toutes ces tracasseries vous ne songez pas à faire un autre métier ?
